# Realizing in-house algorithm-driven free fibula flap set up within 24 hours: a pilot study evaluating accuracy with open-source tools

**DOI:** 10.3389/fsurg.2023.1321217

**Published:** 2023-12-15

**Authors:** Andreas Vollmer, Babak Saravi, Niko Breitenbuecher, Urs Mueller-Richter, Anton Straub, Luka Šimić, Alexander Kübler, Michael Vollmer, Sebastian Gubik, Julian Volland, Stefan Hartmann, Roman C. Brands

**Affiliations:** ^1^ Department of Oral and Maxillofacial Plastic Surgery, University Hospital of Würzburg, Würzburg, Germany; ^2^ Department of Orthopedics and Trauma Surgery, Faculty of Medicine, Medical Center - University of Freiburg, University of Freiburg, Freiburg, Germany; ^3^ Department of Anesthesiology, Perioperative and Pain Medicine, Brigham and Women’s Hospital, Harvard Medical School, Boston, MA,United States; ^4^ Faculty of Electrical Engineering, Computer Science and Information Technology Osijek, Josip Juraj Strossmayer University of Osijek, Osijek, Croatia; ^5^ Department of Oral and Maxillofacial Surgery, Tuebingen University Hospital, Tuebingen, Germany

**Keywords:** mandibular reconstruction, ischemia time, preoperative planning, free osteocutaneous fibula graft, computer-aided design and manufacturing (CAD/CAM)

## Abstract

**Objective:**

This study aims to critically evaluate the effectiveness and accuracy of a time safing and cost-efficient open-source algorithm for in-house planning of mandibular reconstructions using the free osteocutaneous fibula graft. The evaluation focuses on quantifying anatomical accuracy and assessing the impact on ischemia time.

**Methods:**

A pilot study was conducted, including patients who underwent in-house planned computer-aided design and manufacturing (CAD/CAM) of free fibula flaps between 2021 and 2023. Out of all patient cases, we included all with postoperative 3D imaging in the study. The study utilized open-source software tools for the planning step, and three-dimensional (3D) printing techniques. The Hausdorff distance and Dice coefficient metrics were used to evaluate the accuracy of the planning procedure.

**Results:**

The study assessed eight patients (five males and three females, mean age 61.75 ± 3.69 years) with different diagnoses such as osteoradionecrosis and oral squamous cell carcinoma. The average ischemia time was 68.38 ± 27.95 min. For the evaluation of preoperative planning vs. the postoperative outcome, the mean Hausdorff Distance was 1.22 ± 0.40. The Dice Coefficients yielded a mean of 0.77 ± 0.07, suggesting a satisfactory concordance between the planned and postoperative states. Dice Coefficient and Hausdorff Distance revealed significant correlations with ischemia time (Spearman's rho = −0.810, *p* = 0.015 and Spearman's rho = 0.762, *p* = 0.028, respectively). Linear regression models adjusting for disease type further substantiated these findings.

**Conclusions:**

The in-house planning algorithm not only achieved high anatomical accuracy, as reflected by the Dice Coefficients and Hausdorff Distance metrics, but this accuracy also exhibited a significant correlation with reduced ischemia time. This underlines the critical role of meticulous planning in surgical outcomes. Additionally, the algorithm's open-source nature renders it cost-efficient, easy to learn, and broadly applicable, offering promising avenues for enhancing both healthcare affordability and accessibility.

## 1. Introduction

The free osteocutaneous fibula graft described by Taylor in 1979 has become an established standardised procedure for the bony reconstruction of defects in the head and neck region (1). Over time, it has developed a wide range of indications, which include tumor surgery, osteomyelitis and osteonecrosis (1). The planning and execution of these complex operations poses major challenges for surgical teams to achieve optimal outcomes for patients (2).

With advances in medical imaging, diagnostics have become less invasive and also provide significantly more detailed data information. As a result, modern imaging techniques generate high-resolution images. Volume or surface rendering as well as computer algorithms enable a better representation of structural complexity through rotation options or sectional views of the 3D data (3). Numerous scientific publications on planning, based on these imaging modalities, showed immense benefits, such as the reduction of surgery time, ischemia time, improvement of symmetry, bone consolidation and function (4–10). In a comparison of various open-source software tools, Ganry et al. introduced Blender (Blender Foundation and Institute, Amsterdam, The Netherlands) as a promising software tool (6). Such tools offer cost-effectiveness as well as high flexibility in planning procedures (7, 11, 12).

With the increasing establishment of virtual planning procedures, industrial partners have offered solutions for patient-specific surgical guides and osteosynthesis plates. However, complex communication and delivery processes affect cost-effectiveness and workflow optimizations (13). A planning process can take up to two weeks, including several web meetings, reducing the flexibility to respond to dynamic processes in the daily clinic routine (7). In addition, individual planning is to some extent restricted by the industrial partners, limited to the possibilities of the industrial software as well as the proficiency of the engineer.

Open-source software tools in contrast can result in cost savings and the possibility to adapt the code to individual requirements (6). In oral and maxillofacial surgery, the planning of complex surgical interventions using such planning solutions has undergone immense change in recent years (7). Surgeons can incorporate sterilisable 3D models into the surgical process, allowing them to plan and perform surgery more independently (5, 6). In-house planning with self-printed drilling templates from the 3D printers and partially adjustable resection guides such as ReconGuide (KLS Martin Group; Gebrüder Martin GmbH & Co. KG; Tuttlingen, Germany) have excelled in this process (14–16).

Although in-house planning has proven to be effective, its success relies on the accuracy and reliability of the planning tools used. As open-source algorithms have begun to play an increasing role in surgical planning, it is crucial to evaluate the performance thoroughly. The aim of this study is to critically evaluate the effectiveness and accuracy of a simple and cost-efficient open-source algorithm used for in-house planning of mandibular reconstructions. A major advantage of the algorithm is the significant time saving which reduces the entire planning process to one hour. Utilizing quantitative metrics, we compared preoperative plans generated by algorithm to actual postoperative situations. Hence, the study sought to quantify the level of anatomical accuracy achieved, along with assessing the algorithm's impact on ischaemia time by the already mentioned reduction of the overall planning time.

## 2. Materials and methods

### 2.1. Study design

All studies were conducted according to the guidelines of the Declaration of Helsinki and approved by the Ethics Committee of the University of Würzburg under Approval number: 2023071101. In this case series, patients who received an in-house planned computer aided desgingend (CAD) and computer aided manufactured (CAM) free fibula flab within our department in the period 2021–2023 were included. We excluded patients who did not receive postoperative 3D imaging. Age, gender, ischemia time, disease entitiy and the transplant site were extracted as study variables. Ischaemia time was defined as the time from ligation to reanastomosis of the artery.

### 2.2. Digital workflow for in-house CAD/CAM planning

All digital planning steps as well as the analysis were carried out using open-source software, namely Blender version 2.83.20 (Blender Foundation and Instutute, Amsterdam, Netherlands) and 3D Slicer version 5.2.2 (17) (downloaded at http://www.slicer.org). The first step in each planning process began with the extraction of anonymised data sets in the form of DICOM-files (Digital Imaging and Communication in Medicine) from the hospital documentation system. The next step was to create STL-files (standard tessellation language) using 3D Slicer software. In order to optimally prepare the STL-files, the segmentation was first limited to the mandible and then prepared for further processing using the 3D slicer module Surface Wrap Solidify (18). The generation of the file for the fibula and the arterial vessels was done in an analogous way.

**Figure 1:**
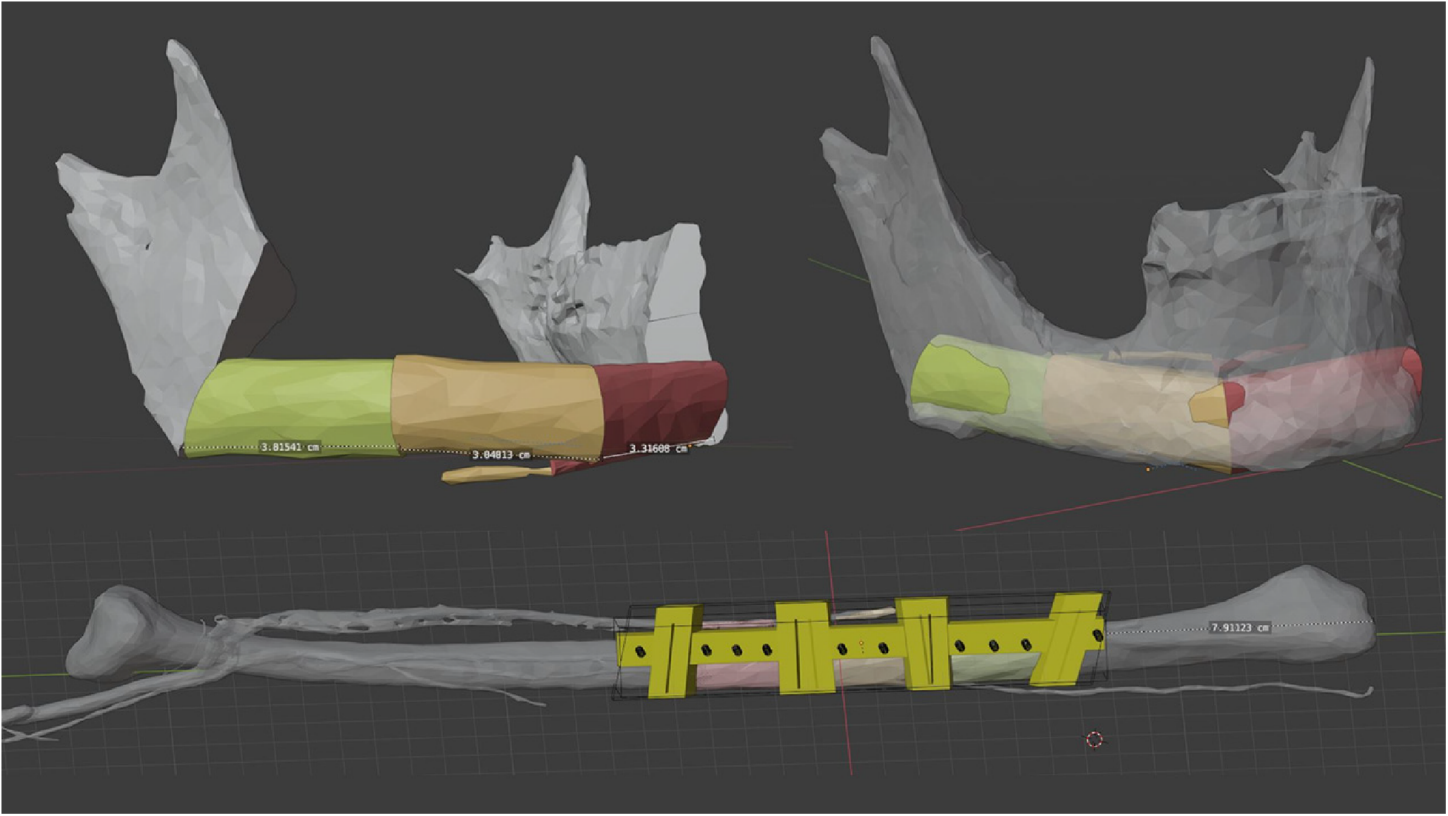
Virtual 3D planning for a osteocutaneous fibula graft using a semi-automated algorithm (19).

Further planning was done with a semi-automated algorithm in the open source software Blender (Figure 1) (19). Only minor steps, such as optimisation of the connector set-up, were carried out manually in order to save time and ensure validation and reproducibility of the workflow.

**Figure 2:**
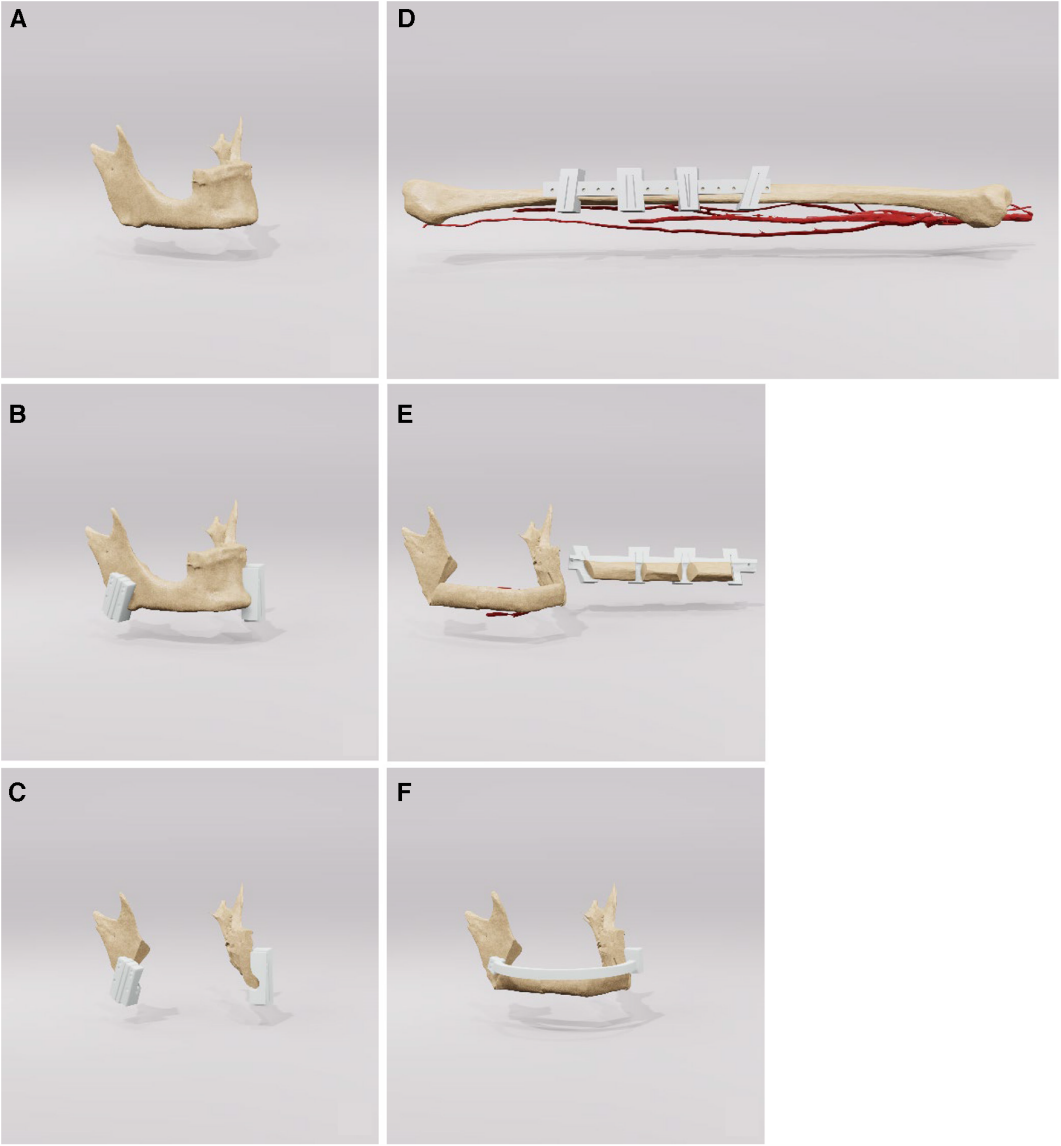
This figure shows a sequence of rendered images used in surgical planning of the fibula. The images go from the initial state (**A**) of the jaw to the jaw (**B, C**) and the fibula with drill guides (**D**) and finally to the reconstructed mandible (**E, F**). These visual aids are an important tool for preoperative discussion and precise surgical planning. surgical planning.

Finally, images were rendered and made available to the surgeons to ensure an optimal preliminary discussion and visualisation of the surgical steps (Figure 2).

### 2.3. Printing and preparation of the drilling templates

The designed drilling templates were then imported into PreForm version 3.30.0 (Formlabs Inc., Somerville, MA, USA) for generating the necessary supporting structures and 3D printing process. The 3D printers used were Form 3 + with the Resin Surgical Guide V1 (Formlabs Inc., Somerville, MA, USA) with a layer thickness of 50 µm. The further processing steps were as specified by the manufacturer, including washing in 99% isopropyl alcohol (IPA) for 20 min followed by a drying phase for at least 30 min. The curing with the form cure were performed at 70°C and 30 min for a Form 3B printer and the Resin Surgical Guide V1. Afterwards, the support structures were removed manually and prepared for autoclaving.

The autoclaving protocol was set according to the guidelines of the American Health Authority in the standard steam autoclaves with a vacuum steam steriliser at 134°C for 4 min and finally with a gravity steriliser at 121°C for a further 30 min. The stencils were then vacuum-sealed and ready for use in the operating theatre.

### 2.4. Surgical workflow

**Figure 3:**
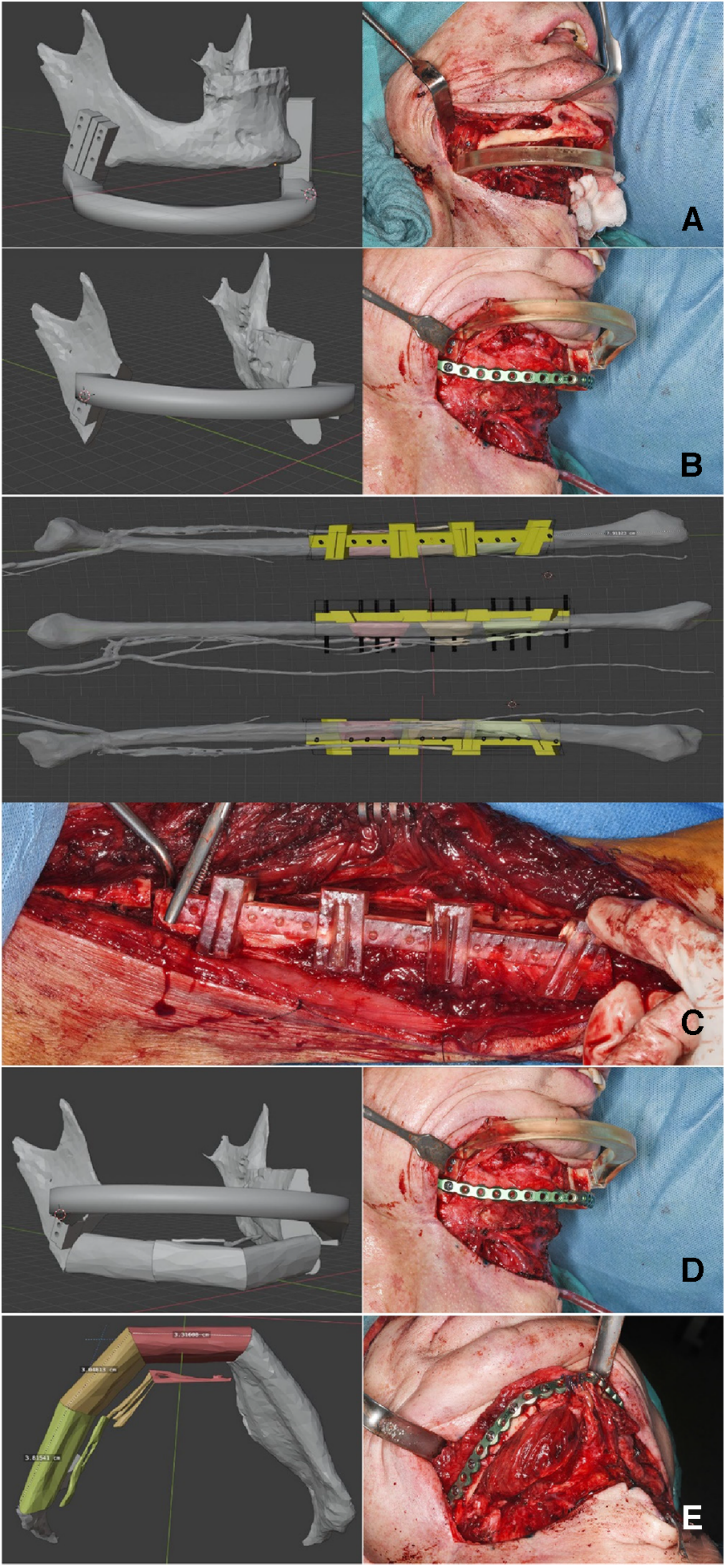
Step-by-step workflow for reconstruction of the mandible. Part **A** outlines the initial resection margins with the cutting guides. Part **B** shows the insertion of a space maintainer and the placement of a pre-contoured osseosynthesis plate. Parts **C, D** describe the osteotomy and the adaptation of the fibula graft, while a virtual osteotomized model is used to check the alignment, Finally, the final surgical result is presented in part **E**.

The surgical intervention employed standard techniques for the reconstruction of the mandible using a free osteocutaneous fibula graft, as per established surgical protocols. Initial resection margins were delineated utilizing cutting guide as illustrated in Figure 3 (part 1). Following this, a placeholder was inserted, featuring drill holes identical to those in the initial cutting guide to guarantee accurate alignment of the temporomandibular joints. Concurrently, a pre-contoured osseosynthesis plate was affixed (part 2). An osteotomy of the free fibula graft was then performed using a custom-made cutting guide (part 3). Steps four and five included the adaptation of the osteotomized fibula graft to the osseosynthesis plate and the placeholder (parts 4 and 5). For validation purposes, a virtual osteotomised model was brought into the operating field to check the removed bony specimen for the accuracy of the planned osteotomy margins. The final step included both the ultimate position check and the final surgical outcome (Figure 3 part 6).

### 2.5. Measurement and three-dimensional analysis of virtual planning and postoperative results

**Figure 4:**
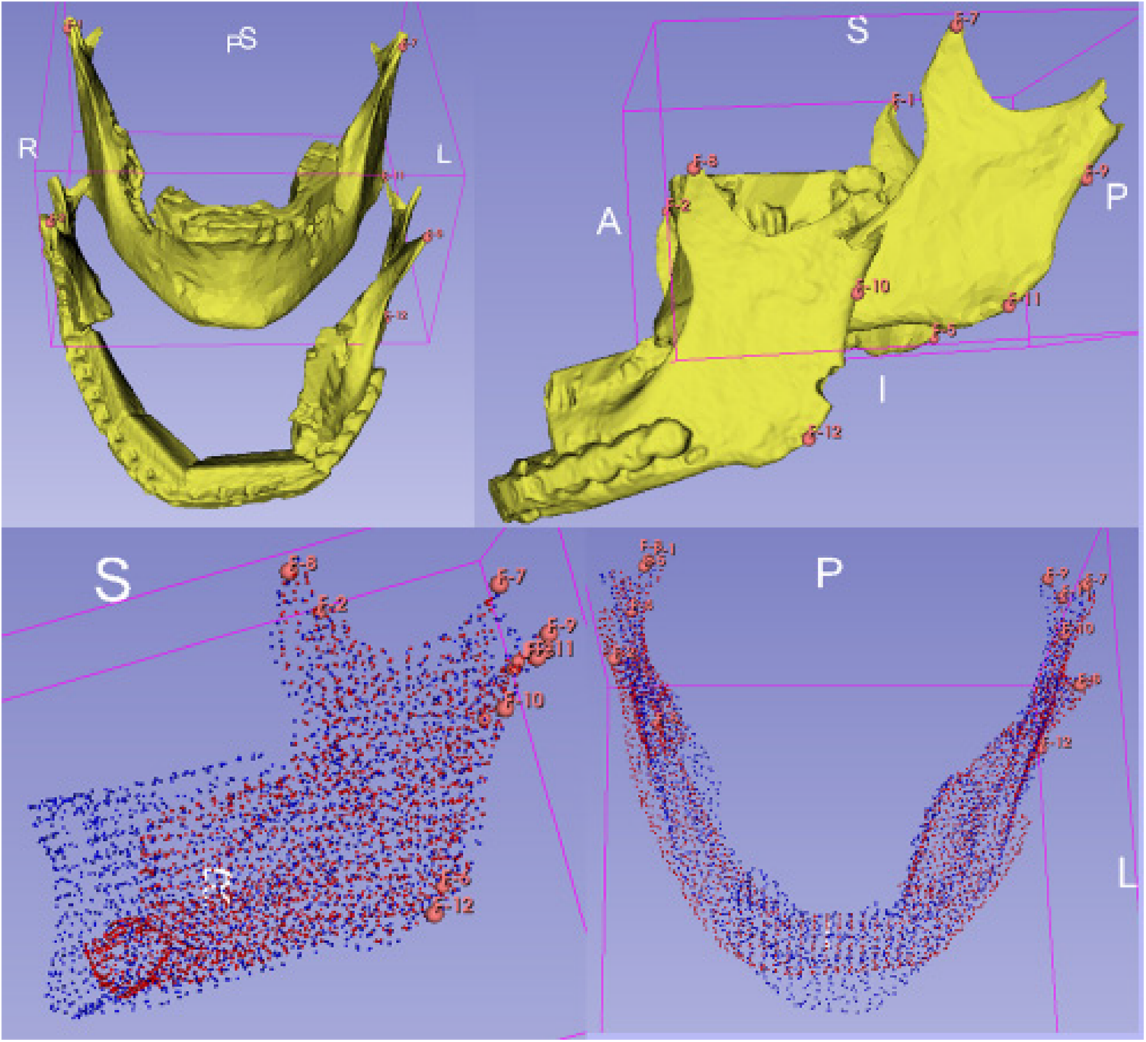
Setting of landmarks, coronoid process, temporomandibular joint and mandibular angle (upper half). Application of the algorithm ALPACA (lower half).

For the analysis of alterations based on defined landmarks, we utilized a module for automatic Landmark Point-based Correspondence Algorithm (ALPACA) in 3D Slicer (20). STL-models of the virtual planning, preoperative situation and postoperative situation reconstructed from the corresponding radiographs were used. The defined landmarks included the coronoid process, the temporomandibular joint and the mandibular angle on both sides respectively (Figure 4).

**Figure 5:**
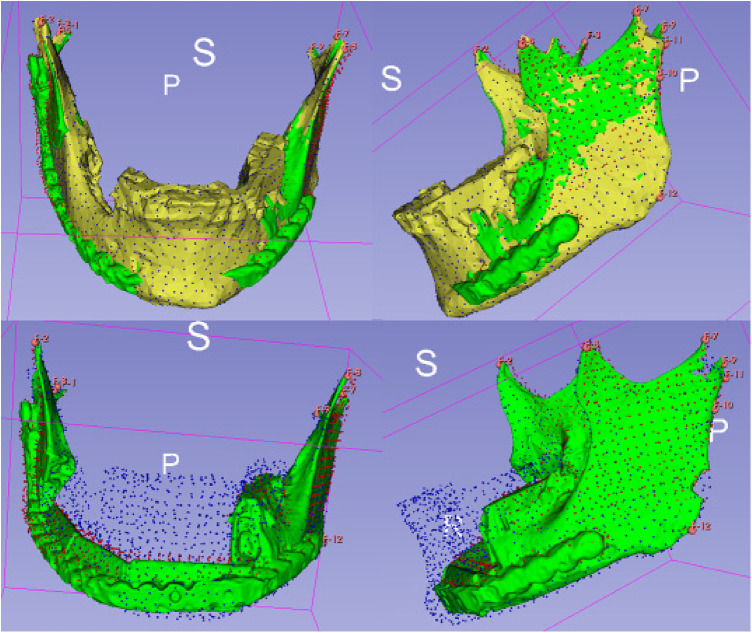
Visualisation of the ALPACA algorithm. Superimposition of the models based on the defined landmarks.

After preparing the dataset as described ALPACA was used to precisely overlay the models (20). ALPACA uses advanced registration and alignment algorithms to achieve accurate superimposition of the models based on the defined landmarks (Figure 5). To ensure the accuracy of the overlay, the point density played an important role. Point density captures the distribution and density of points in the models. A high point density enabled precise alignment of the models and thus contributed to the accuracy of the analysis.

### 2.6. Quantitative evaluation

The 3D slicer segment comparison module was used in this study to allow comparisons between the virtual planning and post-operative situation STL-files. It provides functions for quantitative analysis of segments and evaluation of variation between data sets. Several segments can be analysed at the same time to make a comprehensive evaluation of alterations. We utilized the Hausdorff distance metric and the Dice coeffiicient metric for quantitative analyses (21).

The Hausdorff distance metric is a measure used to quantify the maximum distance between two surfaces or contours. A low Hausdorff distance indicates high agreement or similarity between the models. The Dice similarity metric is a measure of the spatial correspondence between two segmentations. It represents the ratio of the common area of both segments to the total area. Thus a higher Dice score indicates a greater spatial agreement or similarity.

The significance and importance of the Hausdorff distance and the Dice score lie in their ability to provide objective and quantitative information about the accuracy and consistency of segmentations and model registrations. They enable precise assessment of changes between STL-models and contribute to the evaluation of the effectiveness of virtual planning and surgical progress.

### 2.7. Qualitative evaluation

**Figure 6:**
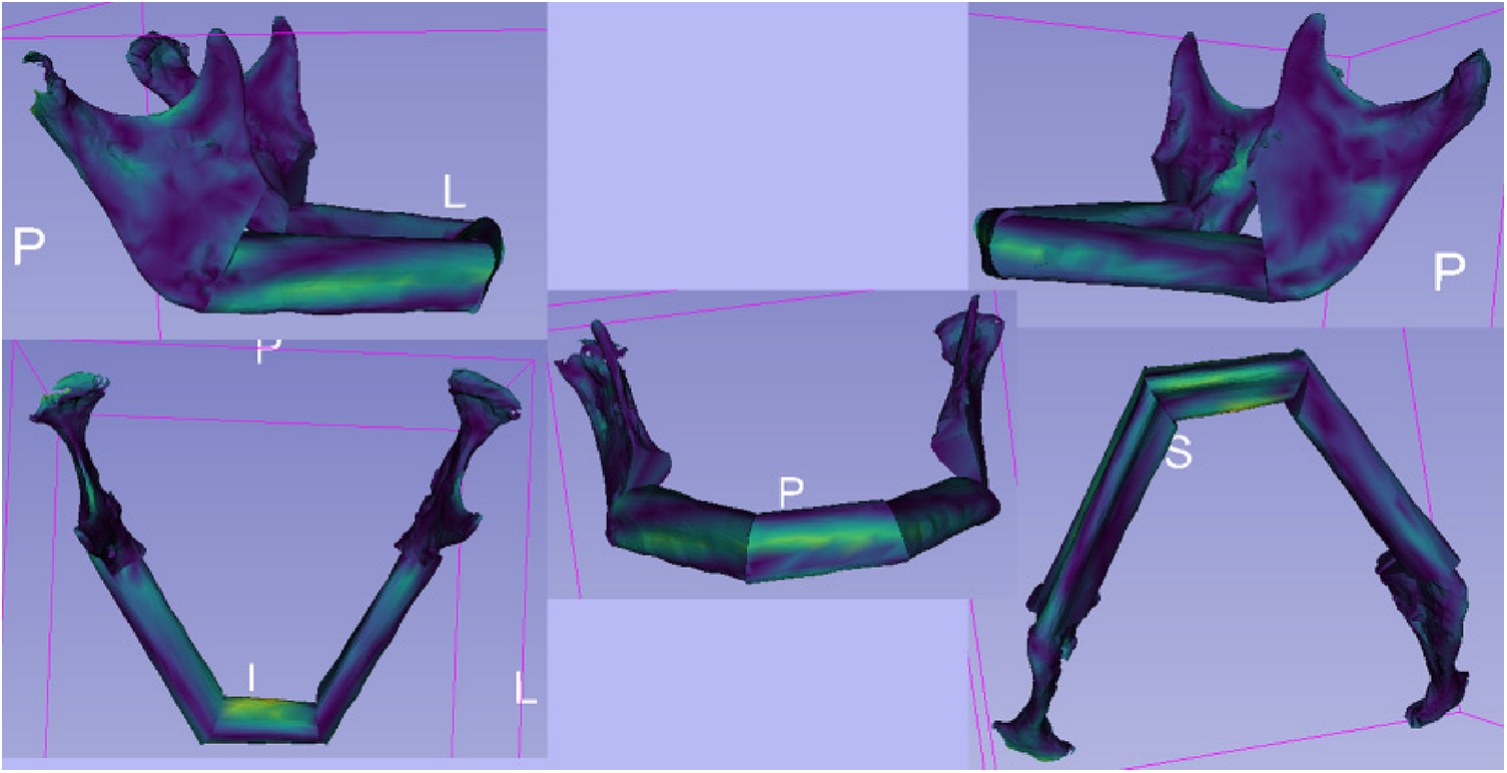
The illustration shows the outcomes of the **“**Model to Model Distance**”** module within the 3D slicer software. The module is designed for precise assessment of spatial differences between two 3D surface models. The visualization depicts color-coded maps highlighting the distances between corresponding points on the two models. These color-coded maps provide insights into areas of minimal and maximal deviation, aiding in the quantitative analysis of shape changes.

For the qualitative comparison of the STL files of the virtual planning and post-operative situation, the module “Model to Model Distance-Absolute Closest Point” (Figure 6) in 3D Slicer was used (22). It allowed the calculation of the distances between the surfaces of the models using the “Absolute Closest Point” method. The distance distribution was visualised and qualitatively evaluated to capture the spatial differences. This contributed to the comprehensive assessment of surgery planning and outcome. In the visualisation, warm colours such as red or yellow were used to indicate larger distances, while cooler colours such as blue or green were used to indicate smaller distances. This allows a more intuitive capture of spatial differences. The colour scheme helped to highlight and visually clarify potential areas of greater variation or need for adjustment between models.

### 2.8. Statistical analyses

Descriptive statistics were performey by calculating mean standard deviations for continous variables and count and relative frequencies (%) for categorical variables. Normality of continous variables was assessed using the Shapiro–Wilk test. Correlation analyses included Spearman rank correlation. Linear regression models were build with ischemia time as dependent variable and the quantitative metrics as well as the disease entitiy as independent variables. The regression coefficient and the 95%-confidence intervall were calculated from the models. A *p*-value of 0.05 was definded as significant. All statistical analyses were coducted in SPSS version 27.0 (IBM Corp., Armonk, NY, USA).

## 3. Results

A total of eight patients were assessed, comprising five males and three females. The age range of the patient cohort spanned from 56 to 66 years, with a mean age of 61.75 ± 3.69 years. The average ischemia time for the cohort was 68.38 ± 27.95 min. The sample included three patients diagnosed with osteoradionecrosis of the jaw, one with medication-related osteonecrosis of the jaw, one with osteomyelitis, and three with oral squamous cell carcinoma (Table 1).

**Table 1:** Descriptive statistics of patients.

		Mean	Standard deviation	Count	Column *N* %
Age		62	4		
Sex	f			3	37.5%
	m			5	62.5%
Disease entity	MRONJ			1	12.5%
	Osteomyelitis			1	12.5%
	ORNJ			3	37.5%
	OSCC			3	37.5%
Transplant	Fibula left			5	62.5%
	Fibula right			3	37.5%

ORNJ, osteoradionecrosis of the jaw; MRONJ, medication-related osteonecrosis of the jaw; OSCC, oral squamous cell carcinoma; f, female; m, male.

For the evaluation of preoperative planning vs. the postoperative outcome, the mean Hausdorff Distance amounted 1.22 ± 0.40. Here we were able to achieve comparable results as in the study by Ritschl et al. (7). The Dice Coefficients yielded a mean of 0.77 ± 0.07, suggesting a satisfactory concordance between the planned and postoperative states.

**Figure 7:**
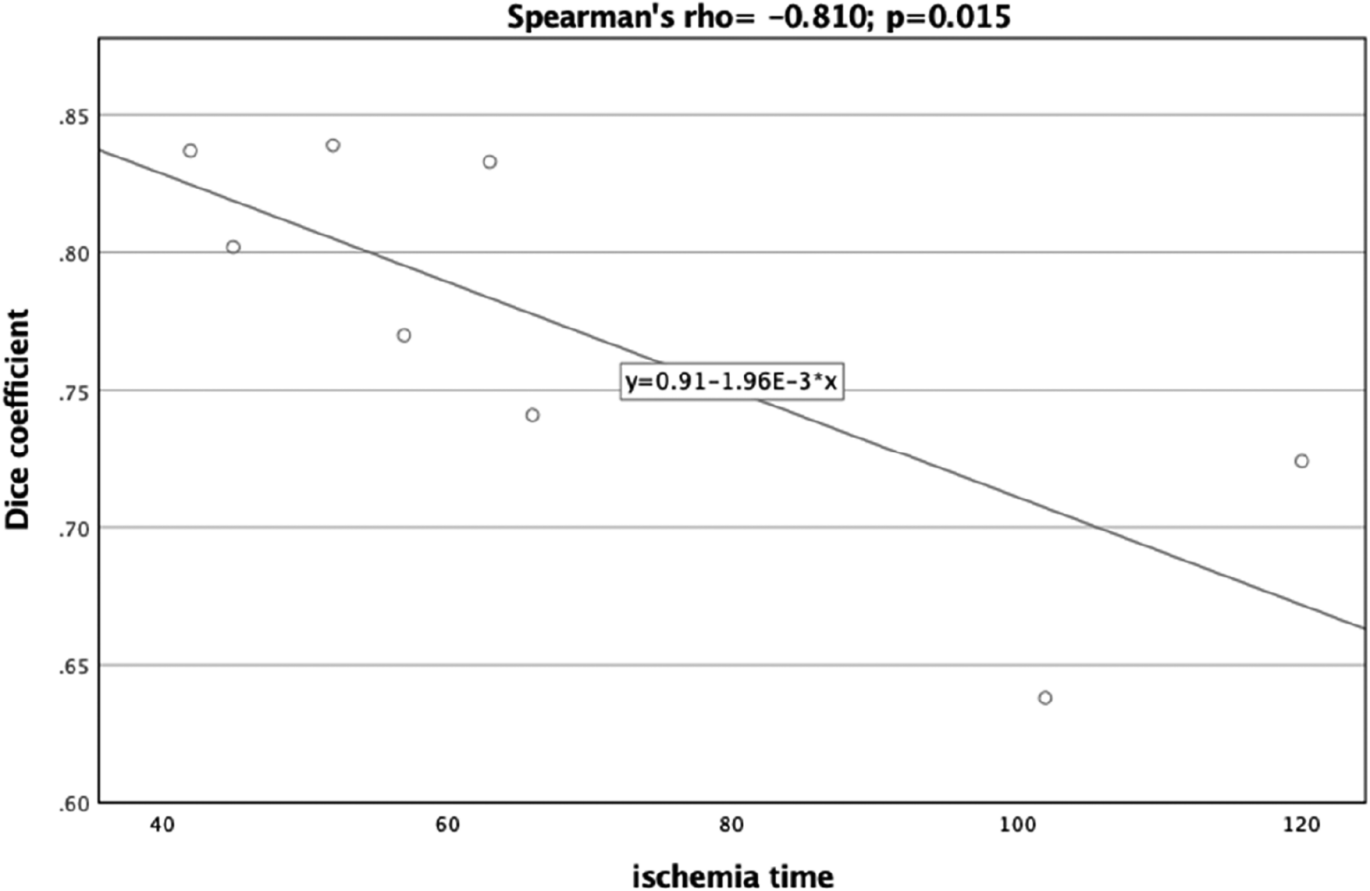
Scatter plot illustrating the relationship between the dice coefficient (planning versus postoperative outcome) and ischemia time.

**Figure 8:**
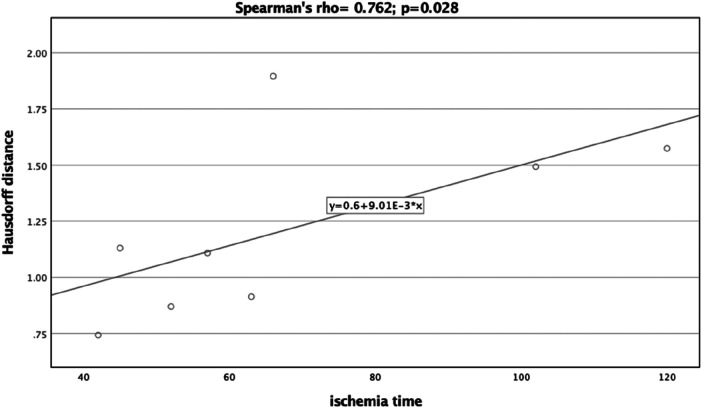
Scatter plot illustrating the relationship between the Hausdorff distance (planning versus postoperative outcome) and ischemia time.

A significant correlation was observed between the Dice Coefficient, assessing the planning vs. postoperative outcome, and ischemia time (Spearman's rho = −0.810, *p *= 0.015) (Figure 7). Additionally, the Hausdorff Distance in the planning vs. postoperative comparison was positively and significantly correlated with ischemia time (Spearman's rho: 0.762, *p *= 0.028) (Figure 8). These results indicate that planning accuracy has a significant influence on ischemia time during surgical procedures.

A linear regression model was utilized to further scrutinize the impact of planning accuracy on ischemia time, while adjusting for disease type. The Dice Coefficient for the planning vs. postoperative comparison had a regression coefficient of −376.39 (95%CI:−634.07,−118.72), *p* = 0.013. Although the regression model for Hausdorff Distance indicated similar trends as the correlation analysis, it did not reach statistical significance [44.25 (95%CI:−18.24,106.74), *p* = 0.128].

## 4. Discussion

This study presents a streamlined, time safing and cost-effective approach for in-house CAD/CAM planning of microvascular free fibula grafts, utilizing an open-source algorithm. Not only does this model significantly reduce the financial burden of such procedures, as corroborated by previous studies (7, 8, 23), but it also eliminates the dependency on industrial partners, thereby enhancing operational flexibility and patient care (7). The application of this algorithm shortens the planning time enormously and thus enables even inexperienced centres to implement the planning in house on a realistic scale. A key advantage lies in the algorithm's ability to generate a virtual blueprint for the surgery, optimizing resection boundaries and vessel localization. Upon setting these parameters, a custom osteosynthesis plate can be preoperatively shaped on the CAD/CAM planned dataset for intraoperative positioning, further reducing surgical time. Through the use of our space maintainer, which has exactly the same drill holes as the initial cutting guide, it is possible for us to secure the exact position of the temporomandibular joints and thus achieve an optimal result of the reconstruction. To the best of our knowledge, there is no study that has analysed this algorithm for its accuracy in terms of final ischaemia time.

An additional benefit is the efficiency and autonomy gained through the in-house planning process. Timely patient care necessitates minimizing the interval between imaging and STL-file preparation, particularly to preserve the chosen surgical margins in the face of potentially growing tumors (24). While this efficiency can restrict intraoperative decision-making if the tumor size exceeds preoperative estimates (23, 25, 26), the presented algorithm reduced planning time to an average of one hour. This marks a significant improvement over reported planning times of 2–3 days (7, 15) or even 3–5 h (26), at other centers. The streamlined approach negates the need for direct communication between surgeons and bioengineers (7, 27). Depending on the material used for template fabrication, additional time may be required. Nevertheless, the presented algorithm enabled us to provide patients with CAD/CAM-planned in-house operations on the same day that imaging is completed.

When comparing a planning file and a postoperative result, limitations with regard to the obtained values for Hausdorff distance measurements and the Dice similarity coefficient must be taken into account. The osteosynthesis plate as an patient specific implant (PSI) is not included in the virtual planning and cannot be projected onto the postoperative result. Furthermore, the osteosynthesis plate leads to typical artefacts which are a general problem in x-ray imaging and ultimately limit an accurate comparison (28).

In the present study, our patient cohort consisted of 8 individuals, predominantly male (62.5%). The majority were diagnosed with osteonecrosis and squamous cell carcinoma, aligning with the expected patient demographics for mandibular reconstructions utilizing microvascular free fibula grafts (29). We employed both the Hausdorff distance and the Dice coefficient to gauge the accuracy between preoperative planning and the final postoperative outcomes. Traditional measurement techniques often neglect the implications of small deviations on the overall three-dimensional geometry of the structure under evaluation. These include point-to-point distances, bone segment lengths, and angles, whose inaccuracies can lead to misalignment in the reconstructed mandible (7, 10). The Hausdorff distance offers a nuanced three-dimensional assessment that captures these small deviations, providing more reliable and precise results (30–32). Similarly, the Dice coefficient provides a robust, scalable metric that is resilient to shape and size variations (33, 34). Our study yielded an average Hausdorff distance of 1.22 ± 0.40, indicative of good agreement as corroborated by previous research (7). The Dice coefficient averaged at 0.77 ± 0.07, confirming satisfactory concordance between preoperative plans and postoperative results. Moreover, both metrics were found to be significantly correlated with ischemia time. The strong negative correlation with the Dice coefficient and the positive correlation with the Hausdorff distance suggest that higher planning fidelity can potentially expedite surgical procedures, thereby reducing ischemia time, as also revealed previously (7, 35, 36). These findings underscore the importance of in-house planning courses for healthcare institutions, as improving planning accuracy could positively affect patient outcomes by minimizing surgical duration (7, 14, 36, 37). Tang et al. conducted a comprehensive evaluation of the benefits and patient-specific outcomes associated with virtual planning. Their study highlighted marked improvements in accuracy, ischemia duration, and overall surgical times, without observing a significant uptick in complications (10). Assessing the efficacy of virtual planning is a complex endeavor, and current literature lacks uniformity in evaluation metrics. Studies have relied on measuring the lengths of fibula segments, distances between specific points, or angles as their primary assessment criteria (7). A recent systematic review examined the impact of virtual surgical planning on fibula free flap-based mandibular reconstruction (12). The study found that virtual surgical planning was associated with a significant reduction in operative time by 44.64 min and indicated a trend toward shorter hospital admission. However, it did not demonstrate statistically significant differences in major or minor complications compared to the traditional techniques. While the review acknowledged reports of high accuracy associated with virtual surgical planning, it noted that insufficient data and the absence of standard measurement criteria are present in the literature (12). Considering the diverse assessments often employed in the literature, which are frequently subjective in nature, we recommend the quantitative approach of utilizing the Dice coefficient and Hausdorff distance metrics for future investigations. This would facilitate more robust and comparable evaluations across different studies.

There are certain limitations to consider. For instance, patient-specific metal artifacts, particularly those associated with dental work, required manual removal from the STL files. Additionally, when large field images were used to align the CBCT with the resection defect, temporomandibular joints were sometimes incompletely imaged, affecting the direct comparability of datasets. Future research should focus on larger cohorts to draw more definitive conclusions.

## 5. Conclusions

In this study, we utilized an open-source, algorithm-driven method for in-house CAD/CAM of microvascular free fibula grafts. Our experience shows that this approach offers not only significant cost advantages but also enhanced flexibility in patient care. Furthermore, the algorithm substantially streamlines planning time, outperforming time frames reported by other medical centers. Assessment of postoperative results using sophisticated evaluation metrics confirmed the high level of accuracy in preoperative planning. Building upon this foundation, structured learning programs for hospitals can make the in-house planning process even more efficient, enabling more reliable and predictable results. Importantly, we observed a significant correlation between planning accuracy and ischemia time, underlining the critical role of meticulous planning in surgical outcomes. Future research could benefit from a larger and more diverse cohort to investigate its potential applications in different clinical settings.

## Data Availability

The original contributions presented in the study are included in the article/Supplementary Materials, further inquiries can be directed to the corresponding author.
